# Evaluation of speech perception in noise in cochlear implanted adults

**DOI:** 10.1016/S1808-8694(15)31195-2

**Published:** 2015-10-20

**Authors:** Leandra Tabanez do Nascimento, Maria Cecília Bevilacqua

**Affiliations:** 1 Master in Human Communication Disorders, Hospital de Reabilitação de Anomalias Craniofaciais (HRAC), USP - Campus Bauru, Ph.D. studies in Special Education under course, Federal University of Sao Carlos (UFSCar), Speech and Hearing Therapist, Centro de Pesquisas Audiológicas (CPA), Hospital de Reabilitação de Anomalias Craniofaciais (HRAC), USP - Campus Bauru.; 2 Full Professor, Department of Speech and Hearing Therapy, Faculdade de Odontologia de Bauru (FOB) - USP - Campus Bauru, Head of the Department of Speech and Hearing Therapy, Faculdade de Odontologia de Bauru (FOB) and coordinator of Centro de Pesquisas Audiológicas (CPA), Hospital de Reabilitação de Anomalias Craniofaciais (HRAC) - USP - Campus Bauru. Centro de Pesquisas Audiológicas (CPA), Hospital de Reabilitação de Anomalias Craniofaciais (HRAC), University of Sao Paulo (USP) - Campus Bauru.

**Keywords:** cochlear implant, speech perception, noise

## Abstract

**Aim**: to evaluate the effects of different signal-to-noise ratios on speech recognition obtained by the use of cochlear implant (CI); to compare the speech recognition in noise with different types of multichannel cochlear implants (CIs) and to evaluate the degree of difficulty for speech understanding in noise in daily life situations. **Study design:** cohort transversal. **Material and Method**: Forty adults with post-lingual hearing loss implanted with Nucleus 22, Nucleus 24, Combi 40, Combi 40+ and Clarion. We evaluated the recognition for CPA sentences in quiet and in S/N +15, +10 and +5 dB. We also applied the Social Hearing Handicap Index (SHHI) questionnaire for self-assessment in daily life. **Results and Conclusion**: All the implanted adults presented a significant reduction in the scores for sentences recognition as the S/N decreased. The medians´ curve for sentence recognition reached 50% in the signal-to-noise ratio of +10 dB. There was no statistically significant difference in sentences’ recognition scores and difficulty scores obtained with the SHHI, for all types of implants. The difficulties of implanted adults were rare in quiet and occasional in noisy situations according to SHHI questionnaire.

## INTRODUCTION

Technological advance has allowed the improvement of strategies for speech signal codification in multichannel cochlear implants. However, the most frequent complaint of patients has been to recognize and understand the speech signal in noise ^1^.

Daily hearing conditions vary enormously concerning the ideal conditions and competitive environmental noise that is frequently found at home, workplace, school, leisure activities and other environments. Implanted patients mention the difficulties to understand in public places, such as in restaurants and parties, or even in a three-people group conversation, when everyone speaks at the same time 2, 3.

The communication in noisy situations has been reported as extremely stressing and orofacial reading is essential in such conditions ^1^.

Explanations for the difficulty to understand speech in noise for patients with sensorineural hearing loss are: noise, that works as masking; loss of binaural integration, which increase the signal/noise ratio in 3dB or more; difficulties in temporal and frequency resolution; reduction of hearing dynamic field and masking effect of low frequency energy about the thresholds of medium and high frequencies, that is, low frequency speech sounds (vowels) are louder and interfere in the perception of high frequency segments (consonants)^4^.

To subjects that use hearing aids, it transmits a processed and amplified acoustic signal to the damaged ear. The integrity of the system after the cochlea is a determining factor in the skills of the users to separate the desired signal from other signals and noise. To subjects that use the cochlear implant, the authors describe that a speech processor codifies the acoustically processed signal to an electrode stimulation pattern. In this case, integrity of the speech processor and its algorithm has been a determining factor for the users’ skills to separate the target signal from other signals ^5^.

The negative influence of noise in speech perception of cochlear implant users may be justified by the following factors: the speech processor codifies the signal to a stimulation pattern of electrodes in quiet that is different from it in noise ^6^; signal processing in the cochlear implant system reduces information and signal redundancy ^3^; monoaural input to the hearing system that consists in one single microphone connected to the speech processor, does not allow processing of noise reduction that is possible in the binaural auditory system 3, 5.

The studies have been carried out to develop new strategies for speech codification ^7^, new noise reducing circuits ^3^, and other supporting devices, such as Beamformer, binaural microphone, which preserves the sound originated from the front and attenuates the sound originated from the side and behind the patient ^5^, modulated frequency systems ^8^, and indication of use of hearing aids in the non-implanted ear ^9^. These technological resources aim at fundamentally promoting improvement in understanding speech in noise.

The report of users of cochlear implant on their performance shows the need to assess speech understanding in competitive noise conditions, that is, in conditions that are close to the reality, in which we have exhibition of different variations in the signal/noise ratio along the day in each environment.

The assessment allows the verification of reduction of cochlear implant user performance in quiet to the competitive noise condition and helps the clinician to indicate and choose technological and therapeutic resources that favor speech understanding of users of cochlear implant in noisy environments.

In addition to analyzing the effectiveness of cochlear implant through the assessment of speech perception in competitive noise, it is necessary to check the level of difficulty of cochlear implant users, in noisy situations of daily life, through self-assessment questionnaires.

The objective of the present study was to assess the effects of different signal/noise ratio, speech recognition with cochlear implant, to compare the recognition of speech in noise, with different types of multichannel cochlear implants; to assess the influence of duration of deafness, use of cochlear implant and progression of deafness in speech recognition with cochlear implant and to assess the level of difficulty that cochlear implant users have in situations with daily competitive noise.

## MATERIAL AND METHOD

The present study was carried out at Centro de Pesquisas Audiológicas (CPA), Hospital de Reabilitação de Anomalias Craniofaciais (HRAC), University of Sao Paulo (USP) - Bauru, and approved by the Research Ethics Committee.

We selected 40 adult patients with post-lingual hearing loss, experience of over 6 months with multichannel cochlear implant use and speech recognition in open-set condition. The subjects were divided into 5 groups (Table 1).

**Table 1:** Characterization of the groups.

Group	Cochlear Implant	Speech Processor	Speech processing	Stimulation mode
G1 (N= 13)	Nucleus 22	Spectra 22	SPEAK	BP+1
G2 (N= 7)	Nucleus 24	Sprint	ACE	MP1+2
G3 (N= 6)	Combi 40	CIS-PRO+	CIS	Monopolar
G4 (N= 7)	Combi 40+	CIS-PRO+	CIS	Monopolar
G5 (N= 7)	Clarion bipolar enhanced 1.2	AB-5200	CIS	Monopolar

Out of 40 studied adults, 19 were male and 21 were female, mean age of 43 years and 6 months at the time of the assessment (median = 44 years and 11 months, ranging from 31 years and 9 months to 62 years and 11 months), mean time of deafness of 5 years and 10 months (median = 2 years and 8 months, ranging from 6 months to 25 years) and mean duration of cochlear implant use of 2 years (median = 1 year and 9 months, ranging from 6 months to 5 years and 11 months).

**Figure 1:**
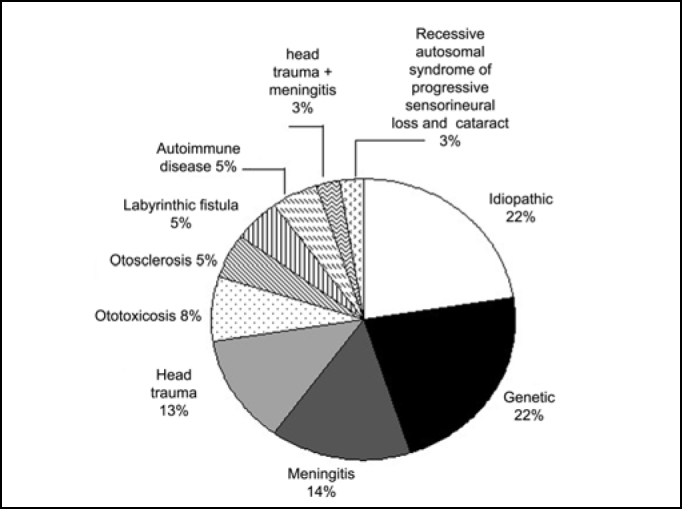
Distribution of subjects concerning etiology of hearing loss.

In Figure 1 we can observe the distribution of subjects concerning etiology of hearing loss. In 20 subjects, hearing loss was progressive and in the others, it was sudden. As to type of insertion, only 1 subject presented partial insertion of electrodes and 2 subjects presented visual impairment.

We carried out CPA sentence recognition test ^10^ recorded in cassette tapes and presented by a two-channel audiometer connected to an amplifier in free field and a loudspeaker, at 0° azimuth, fixed intensity of 70 dB HL, with the subject 1m away from the loudspeaker, in quiet (list 2) and with competitive noise (party noise) ipsilaterally, recorded in a digital compact disc at signal/noise ratio of +15dB (list 3), +10 dB (list 4) and +5 dB (list 5). All procedures were performed in a soundproof booth.

To make self-assessment of the performance of the cochlear implant in daily situations, we used the questionnaire Social Hearing Handicap Index (SHHI)^11^, ^12^ containing 10 questions about auditory skills in quiet situations (hearing loss component) and 10 questions about auditory skills in environment noise (selectivity component).

In the statistical assessment, to compare the groups (different types of cochlear implants) concerning index of CPA sentence recognition in the same hearing situation and scores of difficulty obtained in the same component of the questionnaire SHHI, we used non-parametric Kruskal-Wallis test for independent groups. In the comparison of hearing situations in the same group, we employed the non-parametric test of Friedman for repetitive measurements, and for comparison of scores of difficulty of hearing loss and selectivity components of SHHI questionnaire in the same group, we used non-parametric Wilcoxon test. In all tests, we considered significant result as p<0.05 (5%).

To assess the influence of the characteristics of the subjects (duration of deafness, use of CI and progression of hearing) in CPA sentence recognition index in each situation of hearing, we adjusted the logistic model by using Genmod procedure of statistical software SAS for Windows, version 6.12.

## RESULTS

**Figure 2:**
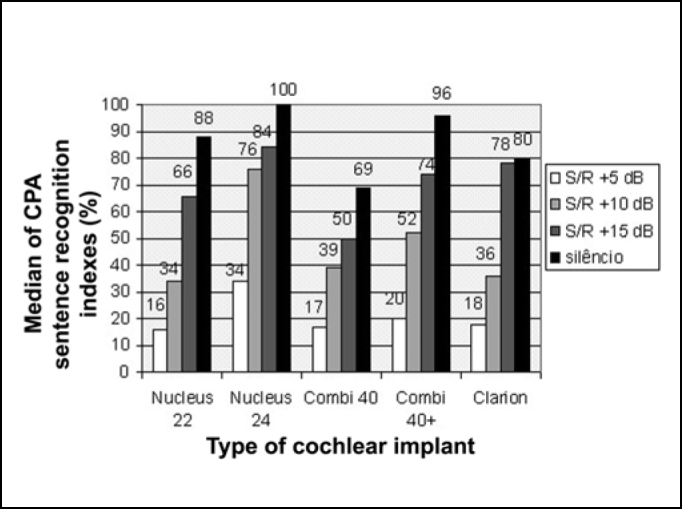
Median of CPA sentence recognition index with cochlear implants Nucleus 22, Nucleus 24, Combi 40, Combi 40+ and Clarion, in situations of S/N ratio of +5 dB, +10 dB, +15 dB and in quiet.

In Figure 2, we observed median of sentence recognition index in situations of S/N of +5 dB, +10 dB, +15 and in quiet with cochlear implants Nucleus 22 (strategy SPEAK), Nucleus 24 (strategy ACE), Combi 40, Combi 40+ and Clarion (strategy CIS).

We can see in Figure 2 a reduction of median of sentence recognition index as a result of reduction of S/N ratio in all types of cochlear implants. The median of sentence recognition index was higher with Nucleus 24 implant (strategy ACE) in all hearing situations.

In the group comparison, Kruskal-Wallis test showed that there was no statistically significant difference (p>0.05) between medians of sentence recognition index obtained for different cochlear implants in all studied hearing situations.

In the comparison between hearing situations in each group, Friedman test revealed that the median of sentence recognition index obtained in quiet was significantly better than that obtained in situations of noise; the median obtained for S/N +15 dB was statistically significant difference better than for the ratios S/N +10 and +5 dB and the median obtained in these S/N ratios was significantly better than in S/N +5 dB.

**Figure 3:**
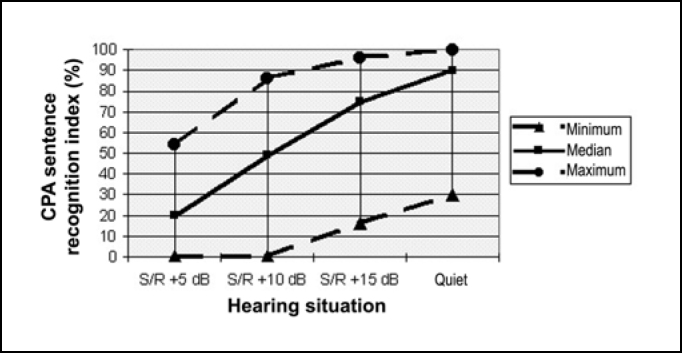
Median, minimum and maximum of CPA sentence recognition indexes in situations of S/N ratio of +5 dB, +10 dB, +15 dB and in quiet with cochlear implant.

In Figure 3, we observed median, minimum and maximum of CPA sentence recognition rates in all hearing situations for the 40 studied subjects, regardless of the type of cochlear implant.

In Figure 3, we visualized a reduction of CPA sentence recognition index as a result of increase in noise levels. The median curve of CPA sentence recognition index reached 50% in the S/N of 10 at 45 dB.

Considering that there was no difference between types of implants and index of sentence recognition, the influence of the characteristics of subjects (duration of deafness, CI duration of use and progression of deafness) in speech perception was independently analyzed from type of cochlear implant.

The statistical analysis revealed that time of deafness had significantly influenced (p<0.05) the rates of sentence recognition indexes in hearing situations of S/N +5 dB, +10 dB and +15 dB, that is, in those situations, the longer the duration of deafness, the lower the CPA sentence recognition index.

The duration of use of cochlear implant significantly influenced (p<0.05) the situations of S/N ratio of +15 dB and in quiet: the longer the use, the higher the rates of CPA sentence recognition in these situations.

The progression of deafness significantly influenced (p<0.05) CPA sentence recognition index in all studied hearing situations. In silence, the subjects with progressive deafness have 1.3719 times more likelihood of getting it correct than sudden deafness cases. In S/N +15, +10 and +5, subjects had with progressive loss had respectively, 1.3356, 2.1876 and 1.2907 more likelihood of getting a correct answer than the subjects with sudden deafness.

**Figure 4:**
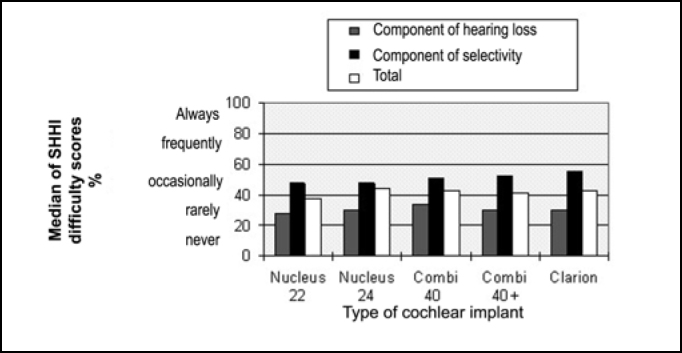
Median of scores of difficulty in Social Hearing Handicap Index (SHHI) in total and in components of hearing loss and selectivity of cochlear implants Nucleus 22, Nucleus 24, Combi 40, Combi 40+ and Clarion.

In Figure 4, we can observe the median of scores of difficulty with hearing loss components, selectivity and total in the SHHI questionnaire with different cochlear implants.

We can see that in Figure 4 users of all types of cochlear implant rarely presented difficulty in quiet, and if present, these difficulties were occasional in the situations that involved selectivity between speech and environment noise.

In the comparison between groups, Kruskal-Wallis test did not show any statistically significant difference between the median of scores of difficulty with SHHI questionnaire in the total and in components between the different types of cochlear implant.

In the comparison of scores of difficulty in the components of SHHI questionnaire in each group, Wilcoxon test indicated that the median scores of difficulty in the components of selectivity were significantly higher (p<0.05) than the component of hearing loss in all types of cochlear implants.

## DISCUSSION

The results obtained in the CPA sentence recognition assessment in the situations of hearing in S/N ratio +5 dB, +10 dB and +15 dB and in quiet, revealed that there was no significant difference between cochlear implant and strategies of speech codification used in this study (Figure 2).

Despite the fact that CIS strategy has high stimulation speed (813 pps per channel in implant Clarion and 1515 pps in implants Combi 40 and Combi 40+), the results of the assessment of speech perception with this strategy were equivalent to the results with strategy SPEAK (250 Hz in implant Nucleus 22) and strategy ACE (Nucleus 24). This last strategy brings together strategy SPEAK and skills of stimulation for strategy CIS, in high speed, emphasizing at the same time, spectral and temporal clues. These results agreed with the findings of previous studies that did not find a difference between sentence recognition in quiet and in noise with the implant Nucleus 22 (strategy SPEAK) and implant Ineraid (strategy CIS)^13^, and between the cochlear implant Nucleus 22 (strategy SPEAK) and cochlear implant Combi 40 (strategy CIS)^2^.

Even though there was no statistically significant difference between cochlear implants, in the results of the speech perception assessment, medians of CPA sentence recognition indexes in S/N ratios of +5 dB, +10 dB and +15 dB, they were higher with cochlear implant Nucleus 24 (strategy ACE) (Figure 2). These results agreed with previous studies that showed that users of cochlear implant Nucleus 24 used strategy ACE and presented better performance than strategies CIS and SPEAK in sentence recognition in quiet and in noise, even though this difference has not been statistically significant 14, 15.

In Figure 2, we could observe that the best performance was obtained in the situation of quiet and the worse in the situation of S/N +5 dB, in all types of cochlear implants used in this study.

The introduction of competitive noise in the test room caused significant reduction of performance to all assessed hearing situations, even in situations considered to be more favorable, with S/N +15 dB, in which speech signal level was 15 dB above the noise level, as detected in previous studies 16, 17.

We could detect a reduction in sentence recognition indexes as a result of increase in levels of noise (Figure 3), with the curve of medians in the CPA sentence recognition index reaching 50% in S/N of +10 dB for users of cochlear implant, and in another study the curve of sentence recognition for cochlear implant users reached 50% in S/N +12.5 dB^18^, whereas for subjects with normal hearing this value was reached in the S/N ratio of -7 dB^19^ (Nascimento 2002).

Subjects’ characteristics such as duration of deafness, duration of CI use and deafness progression, influenced significantly the CPA sentence recognition index. The longer the deafness, the lower the rates of CPA sentence recognition in ratios S/N +5 dB, +10 dB and +15 dB, evidencing that deafness time is one of the most important indicators of post-surgical performance 17, 20.

The longer the duration of implant use, the higher the CPA sentence recognition index in quite and in S/N + 15 dB, because there is significant improvement in recognition and speech understanding as time goes by, especially during the first year 21, 22.

The progression of deafness has significantly influenced CPA sentence recognition indexes, given that in all studied situations subjects with progressive hearing had higher chances of having a correct answer than sudden deafness subjects. It may be justified by the fact that profound sudden deafness causes a drastic change to auditory skills, whereas in progressive hearing loss, auditory skills gradually change, allowing an adaptation, plus a period of benefit with the hearing aid ^22^.

Medians of scores of difficulty obtained in SHHI questionnaire in total and in components of hearing loss and selectivity revealed that there was no statistically significant difference between the used cochlear implants (Figure 4), confirming the results of clinical assessment, which did not evidence statistically significant differences between cochlear implants and speech perception in noise and in ratios S/R +5 dB, +10 dB and +15 dB.

Median of scores of difficulty in selectivity component were significantly higher than in the component of hearing loss, in all types of cochlear implants and difficulties were rare in quiet and occasional in situations that involved environment noise (Figure 4). Findings that agreed with clinical assessment demonstrated worsening in speech recognition when competitive noise was introduced in the test environment.

The main difficulty of cochlear implant users in daily life is to understand speech in noise, regardless of the type of multichannel cochlear implant used ^1^.

It is important to emphasize that high rates of speech recognition and daily performance of cochlear implant users in situations with competitive noise in the study reflected the strict criteria for indication of cochlear implant during pre-surgical assessment ^23^; however, indexes of speech recognition were lower and daily difficulties were higher than for subjects with normal hearing ^19^.

Clinical and subjective assessment of speech perception in competitive noise situations contributes to defining a real profile of the performance of cochlear implant in daily life and, based on this database, the speech therapist can guide patients to use noise suppression systems, available in speech processors, and to indicate technological resources, such as modulated frequency statistics and use of hearing aid in the non-implanted ear. Another possibility is bilateral cochlear implant, which has already proven to improve speech understanding in noise 24, 25.

## CLOSING REMARKS

Cochlear implants have presented significant advances for the past decades relative to speech codification strategies as reported here, but current devices still do not restore normal perception of speech, especially in adverse situations such as presence of noise or many speakers at the same time. New perspectives concerning speech perception are expected based on studies about the combined use of electrical and acoustic stimulation, bilateral cochlear implant and strategies of speech codification that are more similar to the speech processing in the normal cochlea.

To clinically work by directing to the optimization of the speech perception that each cochlear implant user has, it is essential to be familiarized with the scientific and technological knowledge available for the diagnosis of hearing loss, indication and programming of cochlear implant, hearing aids and modulated frequency systems, training of auditory skills, and the strategies of communication associated with knowledge of intervening variables in speech perception.

## CONCLUSION

• Users of cochlear implant presented significant reduction of CPA sentence recognition indexes as a result of reduction in S/N ratio;
• The best CPA sentence recognition index with cochlear implant, in the presence of competitive noise, was S/N of +15 dB, and CPA sentence recognition reached 50% in S/N ratio of +10 dB;
• There were no statistically significant differences in CPA sentence recognition with cochlear implants Nucleus 22 (strategy SPEAK), Nucleus 24 (strategy ACE), Combi 40, Combi 40+ and Clarion (strategy CIS), in quiet and in ratios of N/R +5 dB, +10 dB and +15 dB;
• The longer the duration of deafness, the smaller the CPA sentence recognition index in ratios S/N + 5 dB, +10 dB and +15 dB;
• The longer the use, the higher the CPA sentence recognition indexes in quiet and in S/N ratio + 15 dB;
• The users of cochlear implant with progressive deafness had more chances of correct response than those with sudden deafness, in all situations of assessment;
• The users of cochlear implant presented scores of difficulty that were higher than the component of selectivity (noise) than in the component of hearing loss (quiet) in SHHI questionnaire;
• The difficulties in cochlear implant users were rare in situations of quiet and occasional in situations with daily competitive noise;
• There were no statistically significant differences in scores of SHHI questionnaire with cochlear implants Nucleus 22 (strategy SPEAK), Nucleus 24 (strategy ACE), Combi 40, Combi 40+ and Clarion (strategy CIS).
